# Profile of hospitalizations for neoplasms in the Brazilian Unified Health System: a time-series study

**DOI:** 10.11606/s1518-8787.2021055003192

**Published:** 2021-11-12

**Authors:** Analy da Silva Machado, Anaely da Silva Machado, Dirce Bellezi Guilhem

**Affiliations:** I Universidade de Brasília Faculdade de Ciências da Saúde Programa de pós-graduação em Enfermagem Brasília DF Brasil Universidade de Brasília. Faculdade de Ciências da Saúde. Programa de pós-graduação em Enfermagem. Brasília, DF, Brasil; II Universidade de Brasília Faculdade de Administração, Contabilidade Economia, e Gestão Pública Programa de Pós-Graduação em Economia Brasília DF Brasil Universidade de Brasília. Faculdade de Administração, Contabilidade Economia, e Gestão Pública. Programa de Pós-Graduação em Economia. Brasília, DF, Brasil; III Universidade de Brasília Faculdade de Ciências da Saúde Departamento de Enfermagem Brasília DF Brasil Universidade de Brasília. Faculdade de Ciências da Saúde. Departamento de Enfermagem. Brasília, DF, Brasil

**Keywords:** Neoplasms, Hospitalization, trends, Access to Health Services, trends, Differences in Health Care, Time-series studies

## Abstract

**OBJECTIVE:**

Describe the profile of hospitalizations for cancer diagnosis in Brazil from 2008 to 2018 at Unified Health System (SUS).

**METHODS:**

Time series study of hospitalization rate for malignant neoplasms at SUS. Data were extracted from the Hospital Information System of DataSUS. The trend was estimated using generalized linear regression, applying the Prais-Winsten estimation procedure.

**RESULTS:**

From 2008 to 2018, the hospitalization rate for malignant neoplasms showed an increasing trend at SUS, with an annual variation of 10.7% (p < 0.001; CI = 9.4–11.7). An increasing trend of hospitalizations in all regions of Brazil was observed, except in the Northern region, which remained unchanged. The Northeastern region presented the highest annual variation (13.5%; p < 0.001), whereas the Southern and Southeastern regions had the highest hospitalization rates per 100,000 inhabitants, resulting in 506 and 325 hospitalizations, respectively. We observed a significant increasing trend in hospitalizations of children aged 0 to 9 years (annual variation = 10.9%; p < 0.001); young people, 10 and 19 years (annual variation = 6.9%; p < 0.001); and older adults; over 60 years (annual variation = 7.9%; p < 0.001). Among women, hospitalizations occurred mainly due to malignant neoplasm of the breast (annual variation = 13.2%; p < 0.001); and among men, malignant neoplasm of the prostate (annual variation = 4.7%; p < 0.001).

**CONCLUSION:**

Hospitalizations for malignant neoplasms showed an increasing trend, in line with the increased incidence of cancer, in particular, the most frequent neoplasms between men and women. Although the Northeastern region showed the highest variation in the period, the Southern and Southeastern regions had the highest hospitalization rates in the country. We also observed an increase in hospitalizations among the young (between 0 and 19 years old) and older adults (over 60 years) population. Hospitalizations for neoplasm of the cervix in women, although still the third cause of hospitalizations, showed decreasing behavior.

## INTRODUCTION

Together with cardiovascular, and respiratory diseases and diabetes, cancer is part of the set of chronic non-communicable diseases (NCDs) that cause the most deaths in the world. Cancer reaches individuals in all age groups. When it reaches the economically active young populations^1^, it leads them to disabilities. Currently, cancer ranks second in the ranking of mortality due to NCDs, with an incidence of 24.3 million new cases worldwide in 2017^2,3^.

In biennium 2014–2015, an incidence of 576,000 new cases of cancer per year was estimated in Brazil. Incidences of more than 600,000 and 625,000 new cases of cancer were predicted to occur for the biennium 2018–2019 and 2020–2021, respectively^2,4,5^.

The Unified Health System (SUS), created by Law No. 8,080/1990, ensures universal access to health services at all levels of care and completeness of care in Brazil^6^. To ensure these principles in cancer treatment, Ordinance No. 874 was published in 2013 and established the National Policy for Cancer Prevention and Control in the Health Care Network of People with Chronic Diseases. The establishment of this ordinance aims to reduce mortality and disabilities caused by cancer, and the incidence of some types of cancer by diagnosing early and using screening programs^7^.

Ordinance No. 874 also establishes the criteria for guidance for patients to access health services. In December 2019, the specialized outpatient and hospital care network in oncology at SUS covered 419 services qualified in high oncologic complexity, 9 isolated radiotherapy services, and 21 general hospitals with oncologic surgery^8^. We highlight that clinical hospitalizations also occur in other SUS services.

Clinical treatments, which include chemotherapy and radiotherapy performed on an outpatient basis, are recorded by High Complexity Procedure Authorizations (APAC) and represent the highest percentage of procedures related to cancer treatment in the country. Surgical hospitalizations of cancer patients occur so the performance of biopsies and surgical treatment be done, whereas clinical hospitalizations occur for continuous infusion chemotherapy or the treatment of cancer complications, as in cases of clinical decompensation requiring hospitalization support. The latter can occur in any type of hospital, and not only in specialized ones^9^.

Cancer treatment presents a high cost compared to the other treatments offered by SUS. A study conducted by Barros Reis^10^ showed that the average cost of cancer treatment in Brazil was around US$ 3,796.00 per patient in 2011, and 30% are related to hospitalizations; and the rest, outpatient procedures. In a survey on costs of cervical cancer treatment in public services in 2006, Novaes *et al*.^11^ observed that, of US$ 104,966,045, about 8% were destined for clinical hospitalizations. In a recent study, we observed that the costs of hospitalizations of patients with cervical cancer reached 22.2%^12^.

Evidence suggests that hospitalizations for malignant neoplasms have an important role in cancer treatment and, therefore, studying this theme is relevant. Studying how hospitalizations are managed helps to understand the spatial distribution of these treatments at SUS, identifying whether there is a concentration of hospitalizations or health care voids in the regions of the country. Considering this background, this study analyzes trends and describes the profile of hospitalizations for cancer diagnosis at SUS, in Brazil, between 2008 and 2018.

## METHODS

This is a descriptive time series study conducted using data from the Sistema de Informação Hospitalar (Hospital Information System - SIH) of the SUS Department of Informatics (DATASUS). Data were divided into geographic regions, and frequencies were adjusted for the resident population to obtain the appropriate proportion of individuals in each analyzed group^13,14^.

The SIH, established by Ordinance No. 896/1990 of the Ministry of Health, adopts the Hospital Admission Authorization as an instrument to be used by all public administrators and service providers of the SUS to record and process patient identification data, procedures performed, health professionals involved and hotel structure^15^.

In this study, data extracted from SIH were tabulated in the TabWin program version 4.1.5. The national tables and definition files were obtained from the DataSUS website (http://www2.datasus.gov.br/DATASUS/index.php). The data, extracted in January 2020 and aggregated by year, refer to hospitalizations for malignant neoplasms that occurred in health facilities that were attended by the SUS from January 2008 to December 2018. While this study focuses on hospitalizations for malignant neoplasms, only hospitalizations corresponding to codes C00-C97 were considered, according to Chapter II of the 10th International Classification of Diseases (ICD).

We should highlight that the same patient may have undergone several hospitalizations during the analysis period and that not all cancer patients undergo any hospitalization at SUS. Thus, the proportion of deaths cannot be considered as a mortality rate of hospitalizations, nor as a mortality rate due to cancer at SUS, since not all patients progress to death during hospitalization.

The population data we provided by the Brazilian Institute of Geography and Statistics (IBGE) and were consulted in the statistical table database of the institute (https://sidra.ibge.gov.br/home/pms/brasil).

For the Brazil level, the hospitalization rate was calculated as the ratio among the total number of hospitalizations by the total annual population. For the analysis stratified by complexity (medium and high complexity), type of hospitalization (elective and urgent), the outcome of hospitalization (discharge and death), length of stay, and ICU stay, the ratio was considered between the number of hospitalizations in each stratum and the total population for each year. For sex, age group, and state, the ratio between the hospitalization rate and the population in the same stratum per year was calculated (for example, the incidence rate for females is the ratio between the number of hospitalizations of women and the annual Brazilian female population). Incidence rates were adjusted per 100,000 inhabitants. In the analysis of hospitalizations by ICD codes stratified by sex, only the 20 most frequent ICD codes were considered in 2018 for each sex.

To analyze the trend of the standardized hospitalization rate, the methodology of time series analysis described by Antunes and Cardoso^14^was applied, and the following trend model was estimated for the decade of 2008 to 2018:


$$\log\left({\mathrm{taxa}}_{t}\right)=\beta_{0}+\beta_{1}t+u_{t}$$

Where taxa_*t*_ is the rate of hospitalization for malignant neoplasms in the year *t* and *u* is the error of the regression. The coefficient β_1_ indicates the trend of the time series, so that the estimated value represents the change in log(taxa_*t*_) for each additional year *t*. Thus, if the coefficient β_1_ is positive, the trend of the series is increasing and, if it is negative, the trend is decreasing. The rate transformation allows the trend to be expressed in percentage terms and aims to normalize the distribution and stabilize variance, which is one of the assumptions of the model.

The model was estimated using a generalized linear regression model, applying the Prais-Winsten estimation with robust variance. The method is indicated to adjust the existing serial autocorrelation in time-series analyses to heteroscedasticity and obtain robust statistics. From the robust variance, the appropriate confidence interval and p-value for statistical inference were calculated.

To obtain the trend of the series measured by the average annual variation (VMA) in percentage terms, the following formula was applied^14^:


$$VMA=\left(-1+10\beta^{1}\right)\times 100\%$$

The VMA confidence interval was calculated similarly using the minimum (β_1*min*_) and maximum (β_1*max*_) values obtained from the estimation of the trend model^14^:


$$IC_{95\%}=\left[\left(-1+10\beta^{1\min}\right)\times 100\%.\left(-1+10\beta^{1\max}\right)\times 100\%\right]$$

Finally, we reported whether the estimated annual mean variation was unchanged (p > 0.05), decreasing (p < 0.05 and negative variation) or increasing (p < 0.05 and positive variation) in each stratum evaluated^14^.

The data were analyzed in the statistical program Stata, version 16. Because this study avails itself of secondary data from the public domain, submitting the project for analysis by the Research Ethics Committee was not necessary, according to Resolution of the National Health Council No. 466/2012^16^.

## RESULTS

From January 2008 to December 2018, there were 5,469,895 hospitalizations of patients diagnosed with malignant neoplasms, equivalent to 4.4% of all hospitalizations by SUS in the same period. Regarding the total number of hospitalizations for cancer, 51.8% were female, 79.7% were patients over 40 years of age, and 43.4% were hospitalizations of high complexity. Concerning the type of hospitalization, 51.4% were elective, and 10.2% died. Regarding the length of stay, 50.9% of hospitalizations were up to 3 days, 20.2% between 4 and 6 days, 11.5% between 7 and 9 days, and the others above 10 days of hospitalization (17.2%).

**Figure 1:**
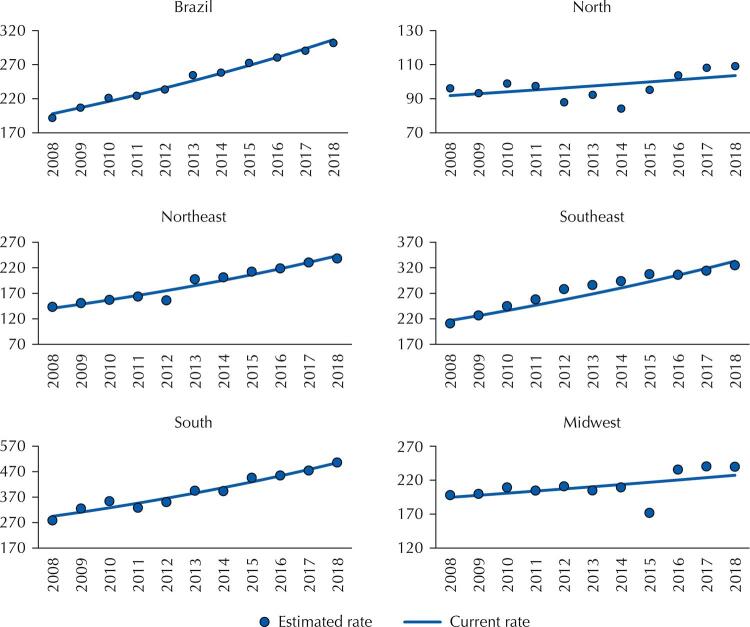
Time series of the rate of hospitalization observed (points) and estimated (line)a for malignant neoplasms (number of hospitalizations per 100,000 inhabitants), per region. Brazil, 2008 to 2018.

^a^ Average annual percentage change of hospitalization rates calculated from the β 1 coefficient of the trend model estimated by generalized linear regression by the Prais-Winsten estimation^13^.

Figure 1 shows the estimated and observed hospitalization rates by region. The Southern region had the highest rate of hospitalizations in the country, with an average of 391 hospitalizations per 100,000 inhabitants per year, followed by the Southeastern (277 per 100,000 inhabitants), Midwestern (211), Northeastern (188), and Northern (97) regions.

**Figure 2:**
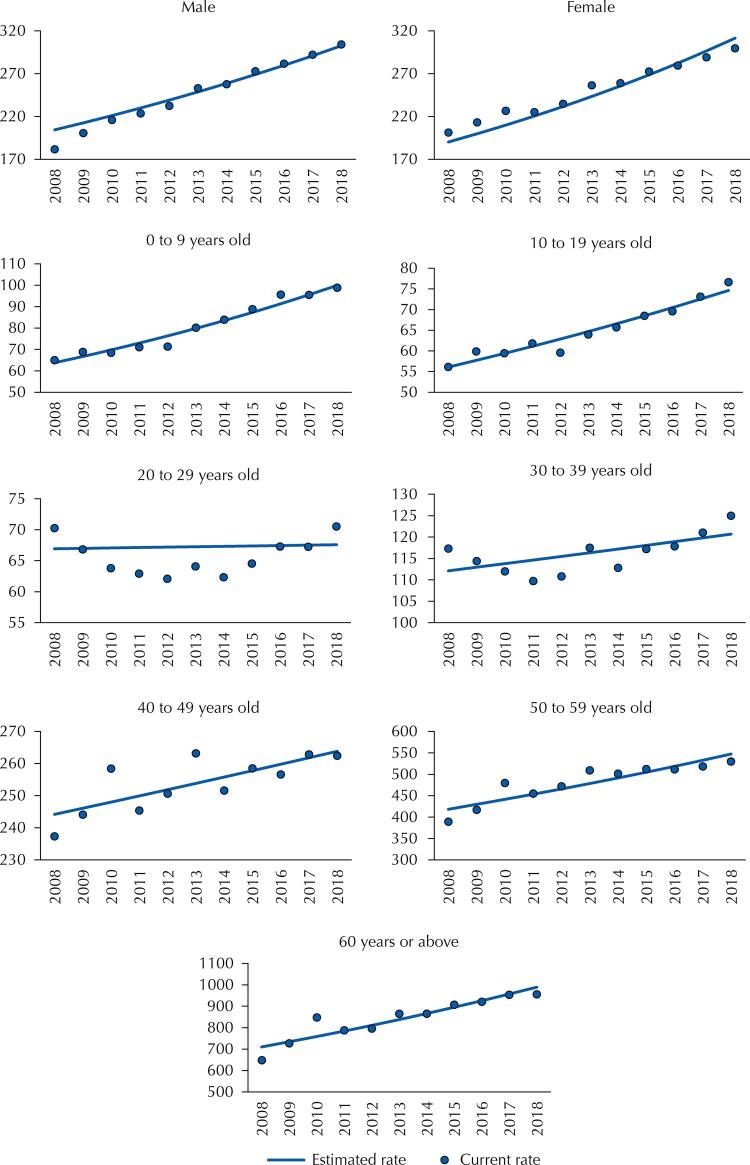
Time series of the rate of hospitalization observed (points) and estimated (line)a for malignant neoplasms (number of hospitalizations per 100,000 inhabitants), by demographic profile. Brazil, 2008 to 2018.

^a^ Average annual percentage change of hospitalization rates calculated from the β 1 coefficient of the trend model estimated by generalized linear regression by the Prais-Winsten estimation^13^.

Figure 2 shows the hospitalization rates observed and estimated by the trend model, according to the population’s demographic profile (sex and age). Figures 1 and 2 together indicate that the actual historical series of the hospitalization rate has a behavior close to that of the series estimated by the linear trend model.

Tables 1 to 3 show the results of the trend analysis for the period 2008 to 2018 and the values of hospitalization rates for malignant neoplasms observed in the initial period (2008) and the final period (2018) of the historical series studied.

Table 1 presents the trend analysis of hospitalizations by region and state. In Brazil, the trend was increasing, with an average annual variation of 10.7% (p < 0.000). An increasing trend in the Northeast (annual variation = 13.5%; p < 0.001), Southeast (annual variation = 10.4%; p < 0.001), South (13.2%; p < 0.001) and Midwest (3.5%, p = 0.043) was observed. Only the Northern region showed an unchanged trend, with an annual change of 2.8% (p = 0.115).

**Table 1:** Hospitalization rate for malignant neoplasms and trend analysis. Brazil, large regions and states, 2008 to 2018.

Region/State	Hospitalization rate for malignant neoplasms (per 100,000 inhabitants)^a^		Trend analysis for the decade 2008–2018			
	
	2008	2018	Annual Percentage Variation	Confidence interval (95%)	p	Trend
Brazil	192	302	10.7	9.4 to 11.7	< 0.001	Increasing
North	96	109	2.8	-0.9 to 6.7	0.115	Unchanged
Rondônia	35	206	60.7	50.7 to 71.4	< 0.001	Increasing
Acre	91	111	4.5	-3.2 to 13	0.223	Unchanged
Amazonas	159	98	-11.1	-16.2 to -5.8	0.001	Decreasing
Roraima	32	143	21.1	-15.3 to 73	0.259	Unchanged
Pará	65	86	6.4	-3.6 to 17.2	0.188	Unchanged
Amapá	68	82	2.3	0.9 to 4	0.006	Increasing
Tocantins	221	158	-6.0	-10.9 to -1.1	0.023	Decreasing
Northeast	143	238	13.5	12.2 to 14.6	< 0.001	Increasing
Maranhão	108	159	10.4	4 to 17.2	0.005	Increasing
Piauí	183	206	3.0	-3.2 to 9.4	0.307	Unchanged
Ceará	166	219	5.4	3.5 to 7.2	< 0.001	Increasing
Rio Grande do Norte	192	377	18.6	14.3 to 23.3	< 0.001	Increasing
Paraíba	136	223	23.9	20.2 to 27.4	< 0.001	Increasing
Pernambuco	183	362	21.6	13.8 to 29.7	< 0.001	Increasing
Alagoas	112	249	18.9	14.6 to 23	< 0.001	Increasing
Sergipe	97	106	1.2	-1.1 to 3.5	0.257	Unchanged
Bahia	118	204	13.2	8.9 to 17.5	< 0.001	Increasing
Southeast	211	325	10.4	7.6 to 13.2	< 0.001	Increasing
Minas Gerais	212	351	12.7	10.4 to 15.1	< 0.001	Increasing
Espírito Santo	201	460	20.8	16.1 to 25.6	< 0.001	Increasing
Rio de Janeiro	172	240	5.7	3.5 to 7.9	< 0.001	Increasing
São Paulo	226	333	8.9	5.7 to 12.5	< 0.001	Increasing
South	279	506	13.2	11.2 to 15.3	< 0.001	Increasing
Paraná	190	553	19.1	7.9 to 31.5	0.003	Increasing
Santa Catarina	269	489	15.1	13.2 to 16.9	< 0.001	Increasing
Rio Grande do Sul	370	468	8.1	2.8 to 13.8	0.007	Increasing
Midwest	198	240	3.5	0.2 to 7.2	0.043	Increasing
Mato Grosso do Sul	216	233	5.2	2.1 to 8.1	0.003	Increasing
Mato Grosso	125	238	20.5	11.7 to 30	< 0.001	Increasing
Goiás	172	202	-1.8	-11.7 to 9.1	0.702	Unchanged
Federal District	325	336	0.2	-10.1 to 11.4	0.980	Unchanged

^a^ Standardized rates by the Brazilian population each year by region, state, and total according to IBGE.

^b^ Average annual percentage change of hospitalization rates calculated from the β 1 coefficient of the trend model estimated by generalized linear regression by the Prais-Winsten estimation^13^.

In 2008, Tocantins had the highest hospitalization rate in the North, but over the years, this rate decreased 6% per year (p = 0.023), while Rondônia and Amapá showed an increasing trend, with annual variations of 60.7% (p < 0.001) and 2.3% (p < 0.001) respectively.

The Northeastern region showed the largest annual percentage change among the five Brazilian regions. Only Sergipe and Piauí showed an unchanged trend within all the states of the region, with annual variations of 1.2% (p = 0.257) and 3% (p = 0.307). Paraíba (annual variation = 23.9%, p < 0.001), Pernambuco (annual variation = 21.6%, p < 0.001), Alagoas (annual variation = 18.9%, p < 0.001) and Rio Grande do Norte (annual variation = 18.6%, p < 0.001) had the most significant mean annual variations observed in the region.

Table 2 shows the rate of hospitalization per profile of patients. Both men and women, the trend was increasing, with annual variations of 11.9% (p < 0.001) and 9.4% (p < 0.001) respectively. We also observed an increasing trend in the age groups from 0 to 9 years (annual variation = 10.9%, p < 0.001), from 10 to 19 years (annual variation = 6.9%, p < 0.001), 40 to 49 years (annual variation = 1.9%; p = 0.001), 50 to 59 years (annual change = 6.4%; p = 0.001) and over 60 years (annual variation = 7.9%; p *<* 0.001). In the age groups between 20 and 39 years, the trend was unchanged. The other groups analyzed showed increasing trends, except for the permanence above 25 days, which was unchanged. No decreasing trend in the analyzed profile in any of the groups studied was observed.

**Table 2:** Hospitalization rate for malignant neoplasms and trend analysis by sex, age group, complexity of the procedure, type of hospitalization, outcome of hospitalization, length of stay, ICU stay, patient origin. Brazil, 2008 to 2018.

Profile of hospitalizations	Hospitalization rate for malignant neoplasms (per 100,000 inhabitants)^a^		Trend analysis for the decade 2008–2018			
	
	2008	2018	Annual Percentage Variation	Confidence interval (95%)	p	Trend
Sex						
Women	201	300	9.4	8.9 to 10.2	< 0.001	Increasing
Men	182	304	11.9	9.9 to 14.3	< 0.001	Increasing
Age group						
0 to 9 years old	65	99	10.9	9.4 to 12.5	< 0.001	Increasing
10 to 19 years old	56	77	6.9	5.7 to 8.1	< 0.001	Increasing
20 to 29 years old	70	71	0.2	-3.4 to 4.0	0.899	Unchanged
30 to 39 years old	117	125	1.6	-0.5 to 4.0	0.121	Unchanged
40 to 49 years old	237	262	1.9	0.9 to 2.6	0.001	Increasing
50 to 59 old	390	530	6.4	3.3 to 9.6	0.001	Increasing
60 years or older	648	956	7.9	4.7 to 11.2	< 0.001	Increasing
Complexity of the procedure						
Mean	121	159	5.7	5.0 to 6.4	< 0.001	Increasing
Discharge	70	142	17.8	15.1 to 20.5	< 0.001	Increasing
Type of hospitalization						
Elective	91	148	11.2	9.6 to 12.5	< 0.001	Increasing
Urgent	100	153	10.2	9.1 to 11.4	< 0.001	Increasing
Outcome of hospitalization						
Discharge	173	271	10.7	9.6 to 11.7	< 0.001	Increasing
Death	19	31	11.2	8.6 to 13.5	< 0.001	Increasing
Length of stay						
Up to 3 days	86	166	15.9	13.2 to 18.3	< 0.001	Increasing
4 to 6 days	46	56	4.7	4.2 to 5	< 0.001	Increasing
7 to 9 days	22	34	10.9	7.6 to 14.3	< 0.001	Increasing
10 to 12 days	11	14	5.0	3.8 to 6.2	< 0.001	Increasing
13 to 15 days	8	10	6.2	5 to 7.4	< 0.001	Increasing
16 to 18 days	5	6	6.9	5.2 to 8.6	< 0.001	Increasing
19 to 21 days	3	4	3.5	1.2 to 5.7	0.007	Increasing
Over 25 days	12	12	0.5	-1.8 to 2.8	0.674	Unchanged
ICU						
No ICU	177	274	10.2	9.1 to 11.2	< 0.001	Increasing
with ICU	15	27	15.1	13.2 to 16.9	< 0.001	Increasing

^a^ Rates standardized by the Brazilian population each year according to IBGE. In the analysis for sex and age group, the rate was standardized by the population according to the same scope. In the analysis for complexity, type of hospitalization, outcome of hospitalization, and permanence, the rate was standardized by the total Brazilian population.

^b^ Average annual percentage change of hospitalization rates calculated from the β 1 coefficient of the trend model estimated by generalized linear regression by the Prais-Winsten estimation^13^.

High complexity hospitalizations presented an annual variation of 17.8% per year (p = 0.001). This fact may be related to the increase in the rate of hospitalizations in which patients needed ICU (15.1% per year, p < 0.001). This data suggest an increase in hospitalizations for surgical purposes when associated with the fact that hospitalizations with a length of stay of up to three days varied by 15.9% per year (p = 0.001).

Table 3 represents the trends of hospitalization for malignant neoplasms in females and males for the 20 ICD codes with the highest hospitalization rates in 2018 for each sex.

**Table 3:** Hospitalization rate for malignant neoplasms and trend analysis. Brazil, 2008 to 2018.

ICD Malignant breast neoplasm	Hospitalization rate for malignant neoplasms (per 100,000 inhabitants)^a^		Trend analysis for the decade 2008–2018			
	
	2008	2018	Annual Percentage Variation	Confidence interval (95%)	p	Trend
Female						
Breast	36.4	62.6	13.2	11.9 to 14.6	< 0.001	Increasing
Colon	11	23	19.1	16.4 to 22.2	< 0.001	Increasing
Cervix	24.4	20.5	-4.7	-7.7 to -1.4	0.011	Decreasing
Other malignant neoplasms of the skin	6.2	19.6	27.9	23.3 to 32.4	< 0.001	Increasing
Ovary	6.8	11.1	12.5	10.2 to 15.1	< 0.001	Increasing
Bronchi and lungs	5	10	18.6	13.8 to 23.6	< 0.001	Increasing
Straight	5	10	19.4	13.2 to 26.2	< 0.001	Increasing
Stomach	5	10	14.3	12.2 to 16.4	< 0.001	Increasing
Thyroid gland	6.8	9.4	7.9	0.9 to 15.3	0.030	Increasing
Connective tissue and other soft tissues	3.4	8.5	22.7	8.4 to 39	0.004	Increasing
Lymphoid leukemia	4.5	8.2	14.6	10.9 to 18	< 0.001	Increasing
Body of the uterus	8.8	7.4	-2.7	-6.7 to 1.6	0.192	Unchanged
Brain	4.7	6.4	6.7	5.2 to 8.1	< 0.001	Increasing
Myeloid leukemia	3.1	5.3	13.8	11.2 to 16.4	< 0.001	Increasing
Pancreas	2	5	25.0	20.8 to 29.4	< 0.001	Increasing
Bladder	2.1	4.5	17.8	13 to 22.7	< 0.001	Increasing
Liver and intrahepatic bile ducts	2	4	20.5	14.6 to 26.8	< 0.001	Increasing
Secondary and unspecified malignancy of the lymph nodes	2.2	4.1	16.1	9.9 to 22.7	< 0.001	Increasing
Esophagus	3	4	5.9	0.2 to 11.9	0.045	Increasing
Malignancy, no location specification	4.3	3.5	-5.4	-8.4 to -2.3	0.003	Decreasing

Male						

Prostate	17.7	32.1	4.7	2.8 to 6.7	< 0.001	Increasing
Colon	11	24	19.1	15.9 to 22.5	< 0.001	Increasing
Other malignant neoplasms of the skin	7.4	22.4	17.2	12.2 to 22.5	< 0.001	Increasing
Stomach	10	19	14.0	11.7 to 16.7	< 0.001	Increasing
Esophagus	9	14	7.9	1.2 to 15.1	0.025	Increasing
Lymphoid leukemia	6.8	13.5	18.9	17.5 to 19.9	< 0.001	Increasing
Bronchi and lungs	9	13	9.9	7.6 to 11.9	< 0.001	Increasing
Straight	5	12	24.2	14.6 to 34.6	< 0.001	Increasing
Bladder	5.4	12	6.2	3 to 9.4	0.001	Increasing
Larynx	7	11	9.1	6.4 to 11.9	< 0.001	Increasing
Connective tissue and other soft tissues	4	9.8	21.1	15.3 to 27.4	< 0.001	Increasing
Brain	5.6	7.6	0.5	-3.8 to 5	0.775	Increasing
Myeloid leukemia	3.9	6.4	15.6	13.8 to 17.2	< 0.001	Increasing
Liver and intrahepatic bile ducts	2	6	25.9	18 to 34.3	< 0.001	Increasing
Pancreas	2	6	24.7	21.1 to 28.8	< 0.001	Increasing
Non-Hodgkin lymphoma	3.3	5.4	4.2	1.4 to 7.2	0.007	Increasing
Secondary and unspecified malignancy of the lymph nodes	2.6	4.9	-4.7	-8.2 to -1.1	0.018	Increasing
Kidney, except renal pelvis	2	4.7	-7.7	-20.6 to 7.4	0.266	Increasing
Oropharynx	2	4	12.2	9.1 to 15.6	< 0.001	Increasing
Malignancy, no location specification	5	4.2	26.2	20.5 to 32.1	< 0.001	Decreasing

^a^ Rates standardized by the Brazilian female population according to IBGE.

^b^ Average annual percentage change of hospitalization rates calculated from the β 1 coefficient of the trend model estimated by generalized linear regression by the Prais-Winsten estimation^13^.

Among women, the most frequent hospitalization rates showed increasing behavior, with significant annual variation. Hospitalizations for malignant breast cancer went from 36.4 per 100,000 women in 2008 to 62.6 per 100,000 women in 2018, with a growth trend of 13.2% per year (p < 0.001). Then, the most frequent hospitalizations in 2018 were for malignant neoplasms of the colon, with 23.1 per 100,000 women (annual variation = 19.1%; p < 0.001), cervix, with 20.5 per 100,000 women (annual variation = −4.7%; p = 0.011), other malignant skin neoplasms, with 19.6 per 100,000 women (annual variation = 27.9%; p < 0.001), and ovary, with 11.1 per 100,000 women (annual variation = 12.5%; p < 0.001).

In men, we observed an increasing trend in the rate of hospitalizations for the main ICD codes. In this group, the main causes of hospitalization were malignant neoplasms of the prostate, with 32.1 per 100,000 men (annual variation = 4.7%, p = 0.001), followed by malignant neoplasms of the colon , with 24 per 100,000 men (annual variation = 19.1%; p < 0.001), other malignant neoplasms of the skin, with 22.4 per 100,000 men (17.2%; p < 0.001), stomach, with 19 per 100,000 men (annual variation = 14%; p < 0.001), and malignant neoplasm of the esophagus, with 14 per 100,000 men (annual variation = 7.9%; p = 0.025).

## DISCUSSION

The analyzed database represents hospitalizations for malignant neoplasms attended by SUS. The frequency of 50.9% of hospitalizations of up to three days refers to surgical hospitalizations or for continuous administration chemotherapy. Longer hospitalizations are usually related to clinical or surgical complications.

Hospitalizations for malignant neoplasms tended to have a significant increase in the country over the period analyzed, with an annual variation of 10.7%. The Northeastern region experienced the largest annual proportional increase in hospitalizations (13.5%), followed by the Southern (13.2%) and Southeastern regions (8.6%). However, the Southern and Southeastern regions had the highest hospitalization rates in 2018, 506 and 325 per 100,000 inhabitants, respectively. These regions have greater availability of services, which increases access to diagnosis, treatment, and hospitalization^17^. For comparison purposes, among 449 establishments qualified in some specialized oncology service, 102 (23%) are located in the South; and 220 (49%), in the Southeast. On the other hand, the lack of care in oncology in the North and Northeast leads patients to seek specialized services in the most assisted regions^18^.

The increase in hospitalizations among children and young people up to 19 years of age should be observed, since, because it occurs less in this age group, there is a lower supply of specialized services for this public. A study by Grabois et al. demonstrated the need to travel long distances so that young people and children receive adequate care^18^. This factor concentrates patients in certain locations, increasing the rate of hospitalizations or treatments in specific regions.

Planning the appropriate distribution of reference centers is important, considering that these are events with lower incidence rates and, therefore, the services will not be made available as often as those related to the most incident neoplasms, even to ensure qualified and specialized care for younger people. For this purpose, health care networks must work in an interconnected way, to direct the patient to the service that offers the indicated treatment.

A study on hospitalizations of children and adolescents with neoplasms in Ribeirão Preto (SP) showed that there are patients from the five regions of the country assisted in the city, which is a reference site for the treatment of onco-hematological diseases with services and specialized staff^19^. The study shows that this concentration of services can interfere with treatment since patients do not always return with adequate frequency due to distance or financial difficulties^19^.

In the study, the highest hospitalization rate among women occurred due to malignant breast cancer, followed by colon neoplasm, corroborating to national estimates that show breast cancer as the most frequent among the female population^2,4,20^.

There is a decreasing trend of hospitalizations due to malignant neoplasms of the cervix. This decline may be the result of the implementation of prevention policies and early diagnosis for this type of cancer. A Study by Arbyn et al.^21^ on cervical cancer demonstrates a global fall in the incidence of this neoplasm, except for some regions in Africa, since there is still difficulty in accessing preventive measures and early diagnosis in less developed regions^22^.

Among men, hospitalizations for malignant neoplasm of the prostate were the most frequent, followed by colon, corroborating national estimates. Malignant neoplasms of the prostate are closely related to age. It is a type of cancer that usually develops slowly, with symptoms that basically affect the genitourinary system and usually lead to hospitalizations^2,4,20,23^.

As for lung cancer, one of the most lethal nowadays, it should be noted that it presented low hospitalization rates among both men and women. This type of cancer, when diagnosed in the early stages, can be surgically resected, but its diagnosis usually occurs in late stages, when only clinical treatments (chemotherapy and radiotherapy) are possible^24,25^, which leads to hospitalizations only in cases of clinical decompensation.

The ICD code of other skin neoplasms appeared as the third cause of hospitalizations among men and the fourth among women. Studies conducted locally already pointed to this occurrence. Most neoplasms that affect the skin are treated at the outpatient level, even when they require surgical treatments. The importance of hospitalization rates due to skin neoplasms draws attention to the fact that life habits, such as protection against ultraviolet radiation, are fundamental to prevent the occurrence of this type of cancer. These data point out the need for prevention programs aimed at sensitizing the population on the subject^26,27^.

The study aimed to outline the profile of hospitalizations due to cancer in patients attended by SUS. We observed that hospitalization rates for cancer increased significantly in the period studied. Data from the Northeastern region, with the highest annual average variation, and the Southern and Southeastern regions, with the highest rates of hospitalization for malignant neoplasms in the country and the highest concentration of specialized resources provided by the SUS. Hospitalizations in age groups below 19 years and above 60 years showed a significant increasing trend in the period. Hospitalizations for malignant neoplasms of the breast were, in isolation, the main cause of hospitalization for cancer among women, whereas malignant neoplasms of the prostate also, in isolation, took the centre stage in hospitalization rate among men.

An important point observed in this study is the existence of health care voids in the most deprived regions, which forces patients to move to distant and specialized centers, often located in other states. It is noteworthy the need for further studies on this subject to be carried out.

Among the limitations of the research, it is worth noting that, as the data represent the number of hospitalizations, it is not possible to infer from them the incidence or mortality rates due to cancer. Not all patients diagnosed with malignant neoplasm are hospitalized, and the same patient may require more than one hospitalization per year, depending on the clinical picture and the evolution of the disease. It is also necessary to consider the cases of neoplasm treated in private care, whose information is not available in the database studied. In addition, it should be considered the fact that the oncological clinical treatment performed through chemotherapy and radiotherapy is processed by the Outpatient Information System (SIA), which was not used in this study.

Another limitation of this study is the absence of analysis of the interactions between the various subgroups analyzed. An example: although the trend for the age group from 20 to 29 years is unchanged, the behavior of the series may be different for men and women in the same subgroup.

Because this is a research conducted with secondary data, there could be a certain limit regarding the reliability of the data presented due to discrepancies in the collection of information or the registration in the SUS systems. However, the Hospital Information System is the official database of the Ministry of Health, and its data support the planning of care and the formulation or adequacy of public policies. Thus, despite possible limitations, the analyzed data can be considered valid.
