# Supplementary material for: Profile of hospitalizations for neoplasms in the Brazilian Unified Health System: a time-series study
Source: Rev Saude Publica. 2021 Nov 12;55:83. doi: 10.11606/s1518-8787.2021055003192 (PMC8687653; doi:10.11606/s1518-8787.2021055003192)
Supplement: Translation

# Translation: Perfil das internações por neoplasias no Sistema Único de Saúde: estudo de séries temporais

**Authors:** Analy da Silva Machado, Anaely da Silva Machado, Dirce Bellezi Guilhem

**Affiliations:** I Brasil Universidade de Brasília. Faculdade de Ciências da Saúde. Programa de pós-graduação em Enfermagem. Brasília, DF, Brasil; II Brasil Universidade de Brasília. Faculdade de Administração, Contabilidade Economia, e Gestão Pública. Programa de Pós-Graduação em Economia. Brasília, DF, Brasil; III Brasil Universidade de Brasília. Faculdade de Ciências da Saúde. Departamento de Enfermagem. Brasília, DF, Brasil

**Keywords:** Neoplasias, Hospitalização, tendências, Acesso aos Serviços de Saúde, tendências, Disparidades em Assistência à Saúde, Estudos de Séries Temporais

## Abstract

**OBJETIVO:**

Descrever o perfil das internações pelo Sistema Único de Saúde (SUS) por diagnóstico de câncer no Brasil no período de 2008 a 2018.

**MÉTODOS:**

Estudo de séries temporais da taxa de internação por neoplasias malignas no SUS. Os dados foram extraídos do Sistema de Informações Hospitalares do DataSUS. A tendência foi estimada por regressão linear generalizada, aplicando o procedimento de Prais-Winsten.

**RESULTADOS:**

No período de 2008 a 2018, a taxa de internação por neoplasias malignas apresentou tendência crescente no SUS, com variação anual de 10,7% (p < 0,001; IC = 9,4–11,7). Observou-se tendência crescente de internações em todas as regiões do Brasil, com exceção da região Norte, que apresentou comportamento estacionário. A região Nordeste foi a que apresentou maior variação anual (13,5%; p < 0,001), enquanto as regiões Sul e Sudeste apresentaram as maiores taxas de internação por 100 mil habitantes, 506 e 325 respectivamente. Observou-se tendência crescente significativa nas internações de crianças de 0 a 9 anos de idade (variação anual = 10,9%; p < 0,001), de jovens entre 10 e 19 anos (variação anual = 6,9%; p < 0,001) e de idosos acima de 60 anos (variação anual = 7,9%; p < 0,001). Entre as mulheres, as internações ocorreram majoritariamente por neoplasia maligna de mama (variação anual = 13,2%; p < 0,001), e entre os homens por neoplasia maligna de próstata (variação anual = 4,7%; p < 0,001).

**CONCLUSÃO:**

As internações por neoplasias malignas mostraram tendência crescente, em consonância com o aumento da incidência de câncer, em particular das neoplasias mais incidentes entre homens e mulheres. Apesar da região Nordeste ter apresentado maior variação no período, as regiões Sul e Sudeste apresentaram as maiores taxas de internação do país. Observou-se também aumento das internações entre a população jovem (entre 0 e 19 anos) e mais idosa (acima de 60 anos). As internações por neoplasias de colo de útero nas mulheres, embora ainda sejam a terceira causa de internações, apresentaram comportamento decrescente.

## INTRODUÇÃO

O câncer, em conjunto com doenças cardiovasculares, respiratórias e diabetes, faz parte do conjunto de doenças crônicas não transmissíveis (DCNT) que mais causam mortes no mundo. Atingindo indivíduos em todas as faixas etárias, o câncer pode levar a incapacidades na população jovem economicamente ativa^1^. Atualmente, o câncer ocupa o segundo lugar no ranking de mortalidade por DCNT, com incidência de 24,3 milhões de casos novos em 2017 no mundo^2,3^.

No biênio 2014–2015 foi estimada uma incidência de 576 mil novos casos de câncer por ano no Brasil. Para os biênios 2018–2019 e 2020–2021, previam-se incidências de mais de 600 mil e 625 mil novos casos de câncer, respectivamente^2,4,5^.

O Sistema Único de Saúde (SUS), criado pela Lei nº 8.080/1990, garante universalidade de acesso aos serviços de saúde em todos os níveis da assistência e a integralidade do cuidado no Brasil^6^. Para garantir esses princípios na assistência oncológica, em 2013 foi publicada a Portaria nº 874, que instituiu a Política Nacional para Prevenção e Controle do Câncer na Rede de Atenção à Saúde das Pessoas com Doenças Crônicas. Essa portaria estabelece como objetivo reduzir a mortalidade e as incapacidades ocasionadas pelo câncer, além de almejar diminuir a incidência de alguns tipos de câncer por meio de programas de rastreamento e diagnóstico precoce^7^.

A Portaria nº 874 também estabelece os critérios para acesso e direcionamento dos pacientes aos serviços de saúde. Em dezembro de 2019, a rede de atenção especializada ambulatorial e hospitalar em oncologia no SUS abrangia 419 serviços habilitados em alta complexidade oncológica, 9 serviços isolados de radioterapia e 21 hospitais gerais com cirurgia oncológica^8^. Cabe notar, ainda, que as internações clínicas também ocorrem nos demais serviços SUS.

Tratamentos clínicos, que incluem quimioterapia e radioterapia realizadas ambulatorialmente, são registrados por Autorizações de Procedimentos de Alta Complexidade (APAC) e representam o maior percentual dos procedimentos relativos a tratamento oncológico no país. As internações cirúrgicas de pacientes oncológicos ocorrem para realizar biópsias e tratamento cirúrgico, enquanto as internações clínicas ocorrem para quimioterapias de infusão contínua ou para tratamento de complicações do câncer, como nos casos das descompensações clínicas com necessidade de suporte pela internação. Essas últimas podem ocorrer em qualquer tipo de hospital, e não apenas nos especializados^9^.

O tratamento oncológico apresenta alto custo em comparação com os demais tratamentos oferecidos pelo SUS. Estudo realizado por Barros Reis^10^ demonstrou que o custo médio do tratamento oncológico no Brasil girou em torno de US$ 3.796,00 por paciente em 2011, sendo que 30% estão relacionados a internações hospitalares, e o restante abrange procedimentos ambulatoriais. Em levantamento sobre custos do tratamento do câncer cervical nos serviços públicos em 2006, Novaes et al.^11^ observaram que, de um montante de US$ 104.966.045, cerca de 8% foram destinados a internações clínicas. Em estudo mais recente, observou-se que os custos com hospitalizações de pacientes com câncer cervical chegaram a 22,2%^12^.

Evidências sugerem que internações por neoplasias malignas têm peso importante no tratamento de câncer e, portanto, estudar essa temática é relevante. O estudo do comportamento das internações ajuda a entender a distribuição espacial desses atendimentos no SUS, identificando se há concentração de internações ou vazios assistenciais em regiões do país. Considerando esse contexto, este estudo analisa tendências e descreve o perfil das internações no SUS por diagnóstico de câncer no Brasil entre 2008 e 2018.

## MÉTODOS

Trata-se de estudo descritivo de séries temporais realizado a partir de dados do Sistema de Informações Hospitalares (SIH) do Departamento de Informática do SUS (DataSUS). Agruparam-se os dados por regiões geográficas, e as frequências foram ajustadas para a população residente a fim de se obter a proporção adequada de indivíduos em cada grupo analisado^13,14^.

O SIH, implantado pela Portaria nº 896/1990 do Ministério da Saúde, adota a Autorização de Internação Hospitalar como instrumento a ser utilizado por todos os gestores e prestadores de serviços do SUS para registrar e processar dados de identificação do paciente, procedimentos realizados, profissionais de saúde envolvidos e estrutura de hotelaria^15^.

No presente estudo, os dados extraídos do SIH foram tabulados no programa TabWin versão 4.1.5. As tabelas nacionais e arquivos de definição foram obtidos no site do DataSUS (http://www2.datasus.gov.br/DATASUS/index.php). Os dados, extraídos em janeiro de 2020 e agregados por ano, referem-se às internações por neoplasias malignas ocorridas nos estabelecimentos de saúde que atenderam pelo SUS no período de janeiro de 2008 a dezembro de 2018. Como o foco deste estudo são as internações por neoplasias malignas, consideraram-se apenas as internações correspondentes aos códigos C00–C97, conforme Capítulo II da 10ª Classificação Internacional de Doenças (CID).

É importante ressaltar que o mesmo paciente pode ter passado por várias internações no período de análise, e que nem todos os pacientes com câncer passam por alguma internação no SUS. Assim, a proporção de óbitos não pode ser considerada como taxa de mortalidade de internações, muito menos como taxa de mortalidade por câncer no SUS, pois nem todos os pacientes evoluem a óbito durante uma internação.

Os dados referentes à população são do Instituto Brasileiro de Geografia e Estatística (IBGE) e foram consultados no banco de tabelas estatísticas do instituto (https://sidra.ibge.gov.br/home/pms/brasil).

Para o nível Brasil, a taxa de internação foi calculada como a razão entre o número total de internações pela população total anual. Para a análise estratificada por complexidade (média e alta complexidade), caráter da internação (eletiva e urgência), desfecho da internação (alta e óbito), dias de permanência e internação na UTI, considerou-se a razão entre o número de internações em cada estrato e a população total para cada ano. Para os recortes de sexo, faixa etária e unidade da Federação, foi calculada a razão entre a taxa de internação e a população no mesmo estrato por ano (por exemplo, a taxa de incidência para o sexo feminino é a razão entre o número de internações de mulheres e a população feminina brasileira anual). As taxas de incidência foram ajustadas por 100 mil habitantes. Na análise das internações por códigos-CID estratificadas por sexo, consideraram-se apenas os 20 códigos-CID mais frequentes em 2018 para cada sexo.

Para analisar a tendência da taxa de internação padronizada, aplicou-se a metodologia de análise de séries de tempo descrita por Antunes e Cardoso^14^, e estimou-se para o período de 2008 a 2018 o seguinte modelo de tendência:


$$\log\left({\mathrm{taxa}}_{t}\right)=\beta_{0}+\beta_{1}t+u_{t}$$

Onde taxa_*t*_ é a taxa de internação por neoplasias malignas no ano *t* e *u* é o erro da regressão. O coeficiente *β*_1_ indica a tendência da série temporal, de modo que o valor estimado representa a mudança em log(taxa_*t*_) para cada ano adicional *t*. Assim, se o coeficiente *β*_1_ é positivo, a tendência da série é crescente e, se for negativo, a tendência é decrescente. A logaritmização da taxa possibilita que a tendência seja expressa em termos percentuais e, adicionalmente, visa normalizar a distribuição e estabilizar a variância, que é uma das suposições do modelo.

O modelo foi estimado por regressão linear generalizada por meio do método de Prais-Winstein com variância robusta. O método é indicado para ajustar a autocorrelação serial existente em análises de séries de tempo e obter estatísticas robustas à heterocedasticidade. A partir da variância robusta, calcula-se o intervalo de confiança e o valor de *p* adequados para a inferência estatística.

Para obter a tendência da série medida pela variação média anual (VMA) em termos percentuais, aplicou-se a seguinte fórmula^14^:


$$VMA=\left(-1+10^{\beta_{1}}\right)\times 100\%$$

O intervalo de confiança da VMA foi calculado de forma similar, utilizando os valores mínimo (β_1min_) e máximo (β_1max_) obtidos da estimação do modelo de tendência^14^:


$$IC_{95\%}=\left[\left(-1+10^{\beta_{1\min}}\right)\times 100\%,\left(-1+10^{\beta_{1\max}}\right)\times 100\%\right]$$

Por fim, reportou-se se a variação média anual estimada foi estacionária (p > 0,05), decrescente (p < 0,05 e variação negativa) ou crescente (p < 0,05 e variação positiva) em cada estrato avaliado^14^.

Os dados foram analisados no programa estatístico Stata, versão 16. Por se tratar de um estudo cuja fonte de informações são dados secundários de domínio público, não houve necessidade de submissão do projeto para análise de Comitê de Ética em Pesquisa, conforme Resolução do Conselho Nacional de Saúde nº 466/ 2012^16^.

## RESULTADOS

No período de janeiro de 2008 a dezembro de 2018, houve 5.469.895 internações de pacientes com diagnóstico de neoplasias malignas, o equivalente a 4,4% de todas as internações pelo SUS no mesmo período. Do total de internações por câncer, 51,8% foram de pessoas do sexo feminino, 79,7% de pacientes acima de 40 anos, e 43,4% eram internações de alta complexidade. Em relação ao caráter da internação, 51,4% foram eletivas, e 10,2% evoluíram para óbito. Quanto ao tempo de permanência, 50,9% das internações foram de até 3 dias, 20,2% de entre 4 e 6 dias, 11,5% de entre 7 e 9 dias, e as demais acima de 10 dias de internação (17,2%).

A Figura 1 apresenta as taxas de internação estimadas e observadas por região. A região Sul apresentou a maior taxa de internações do país, com média de 391 internações a cada 100 mil habitantes por ano, seguida de Sudeste (277 por 100 mil habitantes), Centro-Oeste (211), Nordeste (188) e Norte (97).

**Figura 1:** Série temporal da taxa de internação observada (pontos) e estimada (linha)a por neoplasias malignas (número de internações por 100 mil habitantes), por região. Brasil, 2008 a 2018.

^a^ A taxa de internações estimada foi calculada a partir a partir do coeficiente β_1_ do modelo de tendência estimado por regressão linear generalizada pelo método de Prais-Winsten^13^.

A Figura 2 apresenta as taxas de internação observadas e estimadas pelo modelo de tendência, segundo perfil demográfico da população (sexo e idade). Em conjunto, as Figuras 1 e 2 indicam que as séries históricas reais da taxa de internação têm comportamento próximo ao da série estimada pelo modelo de tendência linear.

**Figura 2:** Série temporal da taxa de internação observada (pontos) e estimada (linha)a por neoplasias malignas (número de internações por 100 mil habitantes), por perfil demográfico. Brasil, 2008 a 2018.

^a^ A taxa de internações estimada foi calculada a partir do coeficiente β_1_ do modelo de tendência estimado por regressão linear generalizada pelo método de Prais-Winsten^13^.

As Tabelas de 1 a 3 apresentam os resultados da análise de tendência para o período de 2008 a 2018 e os valores das taxas de internação por neoplasias malignas observadas no período inicial (2008) e no período final (2018) da série histórica estudada.

A Tabela 1 apresenta a análise de tendência das internações por região e unidade da federação. No Brasil, a tendência foi crescente, com variação média anual de 10,7% (p < 0,000). Também se observou tendência crescente nas regiões Nordeste (variação anual = 13,5%; p < 0,001), Sudeste (variação anual = 10,4%; p < 0,001), Sul (13,2%; p < 0,001) e Centro-Oeste (3,5%, p = 0,043). Apenas o Norte apresentou tendência estacionária, com variação anual de 2,8% (p = 0,115).

**Tabela 1:** Taxa de internação por neoplasias malignas e análise de tendência. Brasil, grandes regiões e estados, 2008 a 2018.

Região/Estado	Taxa de internação por neoplasias malignas (por 100 mil habitantes)^a^		Análise de tendência para o período de 2008 a 2018			
	
	2008	2018	Variação percentual anual^b^	Intervalo de confiança (95%)	p	Tendência
Brasil	192	302	10,7	9,4 a 11,7	< 0,001	Crescente
Norte	96	109	2,8	-0,9 a 6,7	0,115	Estacionário
Rondônia	35	206	60,7	50,7 a 71,4	< 0,001	Crescente
Acre	91	111	4,5	-3,2 a 13	0,223	Estacionário
Amazonas	159	98	-11,1	-16,2 a -5,8	0,001	Decrescente
Roraima	32	143	21,1	-15,3 a 73	0,259	Estacionário
Pará	65	86	6,4	-3,6 a 17,2	0,188	Estacionário
Amapá	68	82	2,3	0,9 a 4	0,006	Crescente
Tocantins	221	158	-6,0	-10,9 a -1,1	0,023	Decrescente
Nordeste	143	238	13,5	12,2 a 14,6	< 0,001	Crescente
Maranhão	108	159	10,4	4 a 17,2	0,005	Crescente
Piauí	183	206	3,0	-3,2 a 9,4	0,307	Estacionário
Ceará	166	219	5,4	3,5 a 7,2	< 0,001	Crescente
Rio Grande do Norte	192	377	18,6	14,3 a 23,3	< 0,001	Crescente
Paraíba	136	223	23,9	20,2 a 27,4	< 0,001	Crescente
Pernambuco	183	362	21,6	13,8 a 29,7	< 0,001	Crescente
Alagoas	112	249	18,9	14,6 a 23	< 0,001	Crescente
Sergipe	97	106	1,2	-1,1 a 3,5	0,257	Estacionário
Bahia	118	204	13,2	8,9 a 17,5	< 0,001	Crescente
Sudeste	211	325	10,4	7,6 a 13,2	< 0,001	Crescente
Minas Gerais	212	351	12,7	10,4 a 15,1	< 0,001	Crescente
Espírito Santo	201	460	20,8	16,1 a 25,6	< 0,001	Crescente
Rio de Janeiro	172	240	5,7	3,5 a 7,9	< 0,001	Crescente
São Paulo	226	333	8,9	5,7 a 12,5	< 0,001	Crescente
Sul	279	506	13,2	11,2 a 15,3	< 0,001	Crescente
Paraná	190	553	19,1	7,9 a 31,5	0,003	Crescente
Santa Catarina	269	489	15,1	13,2 a 16,9	< 0,001	Crescente
Rio Grande do Sul	370	468	8,1	2,8 a 13,8	0,007	Crescente
Centro-Oeste	198	240	3,5	0,2 a 7,2	0,043	Crescente
Mato Grosso do Sul	216	233	5,2	2,1 a 8,1	0,003	Crescente
Mato Grosso	125	238	20,5	11,7 a 30	< 0,001	Crescente
Goiás	172	202	-1,8	-11,7 a 9,1	0,702	Estacionário
Distrito Federal	325	336	0,2	-10,1 a 11,4	0,980	Estacionário

^a^ Taxas padronizadas pela população brasileira de cada ano por região, estado e total de acordo com o IBGE.

^b^ Variação percentual média anual das taxas de internação calculada a partir do coeficiente β_1_ do modelo de tendência estimado por regressão linear generalizada pelo método de Prais-Winsten^13^.

Em 2008, Tocantins tinha a maior taxa de internação do Norte, mas ao longo dos anos essa taxa decresceu 6% por ano (p = 0,023), enquanto Rondônia e Amapá apresentaram tendência crescente, com variações anuais de 60,7% (p < 0,001) e 2,3% (p < 0,001) respectivamente.

O Nordeste apresentou a maior variação percentual anual dentre as cinco regiões brasileiras. Dos estados da região, apenas Sergipe e Piauí apresentaram tendência estacionária, com variações anuais de 1,2% (p = 0,257) e 3% (p = 0,307). Paraíba (variação anual = 23,9%, p < 0,001), Pernambuco (variação anual = 21,6%, p < 0,001), Alagoas (variação anual = 18,9%, p < 0,001) e Rio Grande do Norte (variação anual = 18,6%, p < 0,001) tiveram as variações médias anuais mais significativas observadas na região.

A Tabela 2 mostra a taxa de internação por perfil dos pacientes. Tanto entre homens quanto entre mulheres, a tendência foi crescente, com variações anuais de 11,9% (p < 0,001) e 9,4% (p < 0,001) respectivamente. Observou-se tendência crescente também nas faixas etárias de 0 a 9 anos (variação anual = 10,9%, p < 0,001), de 10 a 19 anos (variação anual = 6,9%, p < 0,001), de 40 a 49 anos (variação anual = 1,9%; p = 0,001), de 50 a 59 anos (variação anual = 6,4%; p = 0,001) e acima de 60 anos (variação anual = 7,9%; p *<* 0,001). Nas faixas etárias entre 20 e 39 anos, a tendência foi estacionária. Os demais recortes analisados apresentaram tendências crescentes, com exceção da permanência acima de 25 dias, que foi estacionária. Não se observou tendência decrescente no perfil analisado em nenhum dos grupos estudados.

**Tabela 2:** Taxa de internação por neoplasias malignas e análise de tendência por sexo, faixa etária, complexidade do procedimento, caráter da internação, desfecho da internação, dias de permanência, internação em UTI, origem do paciente. Brasil, 2008 a 2018.

Perfil das internações	Taxa de internação por neoplasias malignas (por 100 mil habitantes)^a^		Análise de tendência para o período de 2008 a 2018			
	
	2008	2018	Variação percentual anual^b^	Intervalo de confiança (95%)	p	Tendência
Sexo						
Mulheres	201	300	9,4	8,9 a 10,2	< 0,001	Crescente
Homens	182	304	11,9	9,9 a 14,3	< 0,001	Crescente
Faixa etária						
0 a 9 anos	65	99	10,9	9,4 a 12,5	< 0,001	Crescente
10 a 19 anos	56	77	6,9	5,7 a 8,1	< 0,001	Crescente
20 a 29 anos	70	71	0,2	-3,4 a 4,0	0,899	Estacionário
30 a 39 anos	117	125	1,6	-0,5 a 4,0	0,121	Estacionário
40 a 49 anos	237	262	1,9	0,9 a 2,6	0,001	Crescente
50 a 59 anos	390	530	6,4	3,3 a 9,6	0,001	Crescente
60 anos ou mais	648	956	7,9	4,7 a 11,2	< 0,001	Crescente
Complexidade do procedimento						
Média	121	159	5,7	5,0 a 6,4	< 0,001	Crescente
Alta	70	142	17,8	15,1 a 20,5	< 0,001	Crescente
Caráter da internação						
Eletivo	91	148	11,2	9,6 a 12,5	< 0,001	Crescente
Urgência	100	153	10,2	9,1 a 11,4	< 0,001	Crescente
Desfecho da internação						
Alta	173	271	10,7	9,6 a 11,7	< 0,001	Crescente
Óbito	19	31	11,2	8,6 a 13,5	< 0,001	Crescente
Faixa de permanência						
Até 3 dias	86	166	15,9	13,2 a 18,3	< 0,001	Crescente
4 a 6 dias	46	56	4,7	4,2 a 5	< 0,001	Crescente
7 a 9 dias	22	34	10,9	7,6 a 14,3	< 0,001	Crescente
10 a 12 dias	11	14	5,0	3,8 a 6,2	< 0,001	Crescente
13 a 15 dias	8	10	6,2	5 a 7,4	< 0,001	Crescente
16 a 18 dias	5	6	6,9	5,2 a 8,6	< 0,001	Crescente
19 a 21 dias	3	4	3,5	1,2 a 5,7	0,007	Crescente
Acima de 25 dias	12	12	0,5	-1,8 a 2,8	0,674	Estacionário
UTI						
Sem UTI	177	274	10,2	9,1 a 11,2	< 0,001	Crescente
Com UTI	15	27	15,1	13,2 a 16,9	< 0,001	Crescente

^a^ Taxas padronizadas pela população brasileira de cada ano de acordo com o IBGE. Na análise por sexo e faixa etária, a taxa foi padronizada pela população segundo o mesmo recorte. Na análise por complexidade, caráter da internação, desfecho da internação e permanência, a taxa foi padronizada pela população brasileira total.

^b^ Variação percentual média anual das taxas de internação calculada a partir do coeficiente β_1_ do modelo de tendência estimado por regressão linear generalizada pelo método de Prais-Winsten^13^.

As internações de alta complexidade apresentaram variação anual de 17,8% ao ano (p = 0,001). Esse fato pode estar relacionado ao aumento da taxa de internações em que os pacientes precisaram de UTI (15,1% ao ano, p < 0,001). Esse dado, associado ao fato de as internações com permanência de até três dias terem variado 15,9% ao ano (p = 0,001), sugere aumento de internações com finalidades cirúrgicas.

A Tabela 3 representa as tendências de internação por neoplasias malignas no sexo feminino e masculino para os 20 códigos-CID com maiores taxas de internação em 2018, para cada sexo.

**Tabela 3:** Taxa de internação por neoplasias malignas e análise de tendência por diagnóstico principal, por sexo. Brasil, 2008 a 2018.

CID Neoplasia maligna dea	Taxa de internação por neoplasias malignas (por 100 mil habitantes)^a^		Análise de tendência para o período de 2008 a 2018			
	
	2008	2018	Variação percentual anual^b^	Intervalo de confiança (95%)	p	Tendência
SEXO FEMININO						
Mama	36,4	62,6	13,2	11,9 a 14,6	< 0,001	Crescente
Cólon	11	23	19,1	16,4 a 22,2	< 0,001	Crescente
Colo do útero	24,4	20,5	-4,7	-7,7 a -1,4	0,011	Decrescente
Outras neoplasias malignas da pele	6,2	19,6	27,9	23,3 a 32,4	< 0,001	Crescente
Ovário	6,8	11,1	12,5	10,2 a 15,1	< 0,001	Crescente
Brônquios e dos pulmões	5	10	18,6	13,8 a 23,6	< 0,001	Crescente
Reto	5	10	19,4	13,2 a 26,2	< 0,001	Crescente
Estômago	5	10	14,3	12,2 a 16,4	< 0,001	Crescente
Glândula tireoide	6,8	9,4	7,9	0,9 a 15,3	0,030	Crescente
Tecido conjuntivo e de outros tecidos moles	3,4	8,5	22,7	8,4 a 39	0,004	Crescente
Leucemia linfoide	4,5	8,2	14,6	10,9 a 18	< 0,001	Crescente
Corpo do útero	8,8	7,4	-2,7	-6,7 a 1,6	0,192	Estacionário
Encéfalo	4,7	6,4	6,7	5,2 a 8,1	< 0,001	Crescente
Leucemia mieloide	3,1	5,3	13,8	11,2 a 16,4	< 0,001	Crescente
Pâncreas	2	5	25,0	20,8 a 29,4	< 0,001	Crescente
Bexiga	2,1	4,5	17,8	13 a 22,7	< 0,001	Crescente
Fígado e das vias biliares intra-hepáticas	2	4	20,5	14,6 a 26,8	< 0,001	Crescente
Neoplasia maligna secundária e não especificada dos gânglios linfáticos	2,2	4,1	16,1	9,9 a 22,7	< 0,001	Crescente
Esôfago	3	4	5,9	0,2 a 11,9	0,045	Crescente
Neoplasia maligna, sem especificação de localização	4,3	3,5	-5,4	-8,4 a -2,3	0,003	Decrescente

SEXO MASCULINO						

Próstata	17,7	32,1	4,7	2,8 a 6,7	< 0,001	Crescente
Cólon	11	24	19,1	15,9 a 22,5	< 0,001	Crescente
Outras neoplasias malignas da pele	7,4	22,4	17,2	12,2 a 22,5	< 0,001	Crescente
Estômago	10	19	14,0	11,7 a 16,7	< 0,001	Crescente
Esôfago	9	14	7,9	1,2 a 15,1	0,025	Crescente
Leucemia linfoide	6,8	13,5	18,9	17,5 a 19,9	< 0,001	Crescente
Brônquios e dos pulmões	9	13	9,9	7,6 a 11,9	< 0,001	Crescente
Reto	5	12	24,2	14,6 a 34,6	< 0,001	Crescente
Bexiga	5,4	12	6,2	3 a 9,4	0,001	Crescente
Laringe	7	11	9,1	6,4 a 11,9	< 0,001	Crescente
Tecido conjuntivo e de outros tecidos moles	4	9,8	21,1	15,3 a 27,4	< 0,001	Crescente
Encéfalo	5,6	7,6	0,5	-3,8 a 5	0,775	Crescente
Leucemia mieloide	3,9	6,4	15,6	13,8 a 17,2	< 0,001	Crescente
Fígado e das vias biliares intra-hepáticas	2	6	25,9	18 a 34,3	< 0,001	Crescente
Pâncreas	2	6	24,7	21,1 a 28,8	< 0,001	Crescente
Linfoma não Hodgkin difuso	3,3	5,4	4,2	1,4 a 7,2	0,007	Crescente
Neoplasia maligna secundária e não especificada dos gânglios linfáticos	2,6	4,9	-4,7	-8,2 a -1,1	0,018	Crescente
Rim, exceto pelve renal	2	4,7	-7,7	-20,6 a 7,4	0,266	Crescente
Orofaringe	2	4	12,2	9,1 a 15,6	< 0,001	Crescente
Neoplasia maligna, sem especificação de localização	5	4,2	26,2	20,5 a 32,1	< 0,001	Decrescente

^a^ Taxas padronizadas pela população brasileira feminina de acordo com o IBGE.

^b^ Variação percentual média anual das taxas de internação calculada a partir do coeficiente β_1_ do modelo de tendência estimado por regressão linear generalizada pelo método de Prais-Winsten^13^.

Entre as mulheres, as taxas de internação mais frequentes apresentaram comportamento crescente, com variação anual expressiva. As internações por neoplasia maligna de mama foram de 36,4 por 100 mil mulheres em 2008 para 62,6 por 100 mil mulheres em 2018, com tendência de crescimento de 13,2% ao ano (p < 0,001). Em seguida, as internações mais frequentes, em 2018, foram por neoplasias malignas de cólon, com 23,1 por 100 mil mulheres (variação anual = 19,1%; p < 0,001), colo do útero, com 20,5 por 100 mil mulheres (variação anual = −4,7%; p = 0,011), outras neoplasias malignas da pele, com 19,6 por 100 mil mulheres (variação anual = 27,9%; p < 0,001), e ovário, com 11,1 por 100 mil mulheres (variação anual = 12,5%; p < 0,001).

Nos homens, observou-se também tendência crescente na taxa de internações para os principais códigos-CID. Neste grupo, as principais causas de internação foram as neoplasias malignas de próstata, com 32,1 por 100 mil homens (variação anual = 4,7%, p = 0,001), seguidas das neoplasias malignas de cólon, com 24 por 100 mil homens (variação anual = 19,1%; p < 0,001), outras neoplasias malignas de pele, com 22,4 por 100 mil homens (17,2%; p < 0,001), estômago, com 19 por 100 mil homens (variação anual = 14%; p < 0,001), e neoplasia maligna de esôfago, com 14 por 100 mil homens (variação anual = 7,9%; p = 0,025).

## DISCUSSÃO

O banco de dados analisado representa as internações por neoplasias malignas pelo SUS. A frequência de 50,9% de internações de até três dias se refere a internações cirúrgicas ou para realização de quimioterapia de administração contínua. Internações mais longas geralmente estão relacionadas a complicações clínicas ou cirúrgicas.

As internações por neoplasias malignas tiveram tendência de aumento significativo no país ao longo do período analisado, com uma variação anual de 10,7%. A região Nordeste experimentou o maior aumento proporcional anual das internações (13,5%), seguida por Sul (13,2%) e Sudeste (8,6%). Entretanto, as regiões Sul e Sudeste apresentaram as maiores taxas de internação em 2018, 506 e 325 por 100 mil habitantes, respectivamente. Essas regiões contam com maior disponibilidade de serviços, o que aumenta o acesso a diagnóstico, tratamento e internação^17^. Para fins de comparação, dos 449 estabelecimentos habilitados em algum serviço especializado oncológico, 102 (23%) ficam no Sul e 220 (49%) no Sudeste. Por outro lado, a carência assistencial em oncologia no Norte e no Nordeste leva os pacientes a buscar serviços especializados nas regiões mais assistidas^18^.

O aumento das internações entre crianças e jovens de até 19 anos deve ser observado, uma vez que, por se tratar de evento menos incidente nessa faixa etária, há menor oferta de serviços especializados para esse público. Estudo de Grabois et al. demonstrou a necessidade de deslocamento de grandes distâncias para que jovens e crianças recebam assistência adequada^18^. Esse fator concentra os pacientes em determinados locais, elevando a taxa de internações ou tratamentos em regiões específicas.

É necessário, portanto, planejar a distribuição adequada dos centros de referência, considerando que se trata de eventos com menores taxas de incidência e, portanto, os serviços não serão disponibilizados com a mesma frequência daqueles relativos a neoplasias mais incidentes, até mesmo para garantir a assistência qualificada e especializada para os mais jovens. Para tanto, é preciso que as redes de atenção à saúde funcionem de forma interligada, a fim de direcionar o paciente ao serviço que oferta o tratamento indicado.

Estudo sobre internações hospitalares de crianças e adolescentes com neoplasias em Ribeirão Preto (SP) demonstrou que há pacientes das cinco regiões do país assistidos na cidade, que é um local de referência para tratamento de doenças onco-hematológicas, contando com serviços e equipe especializada^19^. O estudo mostra que essa concentração dos serviços pode interferir no tratamento, uma vez que nem sempre os pacientes retornam com a frequência adequada, em virtude da distância ou de dificuldades financeiras^19^.

Na presente pesquisa, a maior taxa de internação entre as mulheres ocorreu por neoplasia maligna de mama, seguida da neoplasia de cólon, corroborando estimativas nacionais que demostram o câncer de mama como o mais incidente entre a população feminina^2,4,20^.

Observa-se tendência decrescente das internações por neoplasias malignas de colo do útero. Esse declínio pode ser resultado da implementação de políticas de prevenção e diagnóstico precoce para esse tipo de câncer. Estudo de Arbyn et al.^21^ sobre câncer de colo do útero demonstra uma queda global na incidência dessa neoplasia, com exceção de algumas regiões na África, uma vez que ainda há dificuldade de acesso a medidas preventivas e diagnóstico precoce em regiões menos desenvolvidas^22^.

Dentre os homens, as internações por neoplasia maligna de próstata foram as mais incidentes, seguidas das de cólon, corroborando estimativas nacionais. As neoplasias malignas de próstata estão intimamente relacionadas com a idade. Trata-se de um tipo de câncer que geralmente se desenvolve de forma lenta, com sintomas que acometem basicamente o aparelho geniturinário e costumam levar a internações^2,4,20,23^.

Quanto ao câncer de pulmão, um dos mais letais na atualidade, cabe observar que ele apresentou baixas taxas de internação tanto entre homens quanto entre mulheres. Esse tipo de câncer, quando diagnosticado nas fases iniciais, pode ser ressecado cirurgicamente, mas seu diagnóstico geralmente ocorre em fases tardias, quando apenas tratamentos clínicos (quimioterapia e radioterapia) são possíveis^24,25^, o que leva a internações somente nos casos de descompensação clínica.

O código-CID de outras neoplasias de pele apareceu como a terceira causa de internações entre os homens e a quarta entre as mulheres. Estudos realizados localmente já apontavam para essa ocorrência. A maioria das neoplasias que acometem a pele são tratadas em nível ambulatorial, mesmo quando demandam tratamentos cirúrgicos. A importância das taxas de internação por neoplasias de pele chama a atenção para o fato de que hábitos de vida, como a proteção contra a radiação ultravioleta, são fundamentais para evitar a ocorrência desse tipo de câncer. Esses dados apontam para a necessidade de programas de prevenção voltados a sensibilizar a população sobre o assunto^26,27^.

O presente estudo teve como objetivo delinear o perfil das internações em decorrência do câncer em pacientes assistidos pelo SUS. Observou-se que as taxas de internação por câncer aumentaram significativamente no período estudado. Destacam-se os dados da região Nordeste, com a maior variação média anual, e das regiões Sul e Sudeste, com as maiores taxas de internação por neoplasias malignas do país e a maior concentração de recursos especializados pelo SUS. As internações em faixas etárias inferiores a 19 anos e acima dos 60 anos mostraram tendência crescente significativa no período. As internações por neoplasias malignas de mama foram, de forma isolada, a principal causa de internação por câncer entre mulheres, enquanto a neoplasia maligna de próstata ocupou, também de forma isolada, a primeira posição em taxa de internação entre homens.

Um ponto importante observado por este estudo é a existência de vazios assistenciais nas regiões mais carentes, o que obriga os pacientes a se deslocarem para polos especializados distantes, muitas vezes localizados em outros estados. Destaca-se a necessidade de que mais estudos sobre esse assunto sejam realizados.

Dentre as limitações da pesquisa, cabe notar que, como os dados representam o número de internações hospitalares, não é possível inferir a partir deles as taxas de incidência ou de mortalidade por câncer. Nem todos os pacientes diagnosticados com neoplasia maligna são internados, e um mesmo paciente pode necessitar de mais de uma internação por ano, a depender do quadro clínico e da evolução da doença. É preciso considerar também os casos de neoplasia atendidos na assistência privada, cujas informações não estão disponíveis na base de dados estudada. Além disso, deve-se considerar o fato de que o tratamento clínico oncológico realizado por meio de quimioterapias e radioterapias é processado pelo Sistema de Informações Ambulatoriais (SIA), que não foi usado neste estudo.

Outra limitação deste estudo é a ausência de análise das interações entre os diversos subgrupos analisados. Um exemplo: embora a tendência para a faixa etária de 20 a 29 anos seja estacionária, é possível que o comportamento da série seja diferente para homens e mulheres no mesmo subgrupo.

Por se tratar de pesquisa realizada com dados secundários, poderia haver certa limitação quanto à fidedignidade dos dados apresentados em razão de discrepâncias na coleta das informações ou no registro nos sistemas do SUS. No entanto, o Sistema de Informações Hospitalares é a base de dados oficial do Ministério da Saúde, e seus dados embasam o planejamento assistencial e a formulação ou adequação de políticas públicas. Dessa forma, apesar de possíveis limitações, pode-se considerar como válidos os dados analisados.
